# Health and behavioral factors associated with binge drinking among university students in nine ASEAN countries

**DOI:** 10.1186/s13011-017-0117-2

**Published:** 2017-06-26

**Authors:** Siyan Yi, Chanrith Ngin, Karl Peltzer, Supa Pengpid

**Affiliations:** 1 KHANA Center for Population Health Research, Phnom Penh, Cambodia; 2 Center for Global Health Research, Touro University California, Vallejo, USA; 3 ASEAN Institute for Health Development, Mahidol University, Bangkok, Thailand; 4 Department of Research & Innovation, University of Limpopo, Sovenga, South Africa; 5 HIV/AIDS/STIs/and TB (HAST), Human Sciences Research Council, Pretoria, South Africa

**Keywords:** Binge drinking, Social and health risk factors, Substance use, University students, ASEAN

## Abstract

**Background:**

Heavy drinking among university students has been globally recognized as a major public health burden. In the Association of Southeast Asian Nations (ASEAN) region, studies on this issue have been scant, country-specific and in different time frames. The aim of this study was to identify social and behavioral factors associated with binge drinking among university students in nine ASEAN countries.

**Methods:**

This cross-sectional study was conducted in 2015 among 8809 undergraduate university students from 13 universities in Cambodia, Indonesia, Laos, Malaysia, Myanmar, the Philippines, Singapore, Thailand and Vietnam using self-administered questionnaire. Multivariate logistic regression analyses were conducted to explore the associated factors.

**Results:**

More than half (62.3%) of the study sample were female with a mean age of 20.5 (SD = 2.0) years. Of total, 12.8% were infrequent (<once per month) and 6.4% frequent (≥ once per month) binge drinkers. After adjustment, among males, higher binge drinking remained significantly associated with being in older age groups, living with parents or guardians, lower level of non-organized religious activity, lack of knowledge on alcohol-heart disease relationship, weak beliefs in the importance of limiting alcohol use, poor subjective health status, lower level of life satisfaction, tobacco and illicit drug use, depressive symptoms and high level physical activity. Among females, higher prevalence of binge drinking remained significantly associated with being in the older age groups, poorer family background, living in an upper-middle- or high-income country, lower level of non-organized religious activity, lack of knowledge on alcohol-heart disease relationship, lack of knowledge on alcohol-high blood pressure relationship, weak beliefs in the importance of limiting alcohol use, lower level of life satisfaction, use of other substances such as tobacco and illicit drug, depressive symptoms and high level of physical activity.

**Conclusions:**

Findings from this study indicate a need for devising or refining university health promotion programs that integrate binge drinking, other substance use, co-occurring addictive behaviors and health beliefs in the respective countries.

## Background

According to the Global Status Report on Alcohol and Health 2014, globally, pure alcohol consumption per person aged 15 years or older was 6.2 l in 2010 [1]. About 16.0% of drinkers aged 15 years or older engaged in heavy episodic drinking. In 2010, the South-East Asia region had an alcohol per capita consumption among people aged 15 years and older of 3.4 l, while the prevalence of heavy episodic drinking was 12.4% among drinkers aged 15 years and older. In the Asia-Pacific region, per capita alcohol consumption averaged 29 l in 2013, compared with 59 l in Europe and 48 l in the rest of the world [2]. In 2010, the prevalence of alcohol use disorders was 2.2%, and that of alcohol dependence was 1.7% in the region. In 2012, about 3.3 million deaths, or 5.9% of all global deaths, stemming from more than 200 diseases and injury conditions, were related to alcohol consumption [1]. The World Health Organization (WHO) called for a 10% reduction in the harmful use of alcohol by 2025 from 2010 levels [1]. Harmful use of alcohol causes not only health, but also social and economic problems, particularly among youth.

Key factors affecting alcohol consumption and alcohol-related harm comprise age, gender, familial risk factors, socioeconomic status, economic development, culture and context and alcohol control and regulation [1]. Further, genetic differences in enzymes influence drinking patterns and explain variations of alcohol-related problems among ethnic groups. Studies show that Asians have defective genes responding to alcohol consumption with intense flushing and other unpleasant reactions [3, 4]. Consequently, Asians consume very little alcohol and are at a much lower risk for alcoholism than Europeans and African Americans having functional genes.

Specifically, heavy drinking among university students has been identified as a major public health burden, mainly based on North American and European samples [5–11]. Few studies including university students in low, middle-income and emerging economy countries indicate that the prevalence of hazardous alcohol use in Australasia, Europe and South America appears similar to that in North America, but lower in Africa and Asia [12, 13]. Heavy drinking was found among male and female university students in Columbia (46% and 24%, respectively) [5], China (16.7%–25.5% and 5.4%–9.1%) [14, 15], Malawi (54.1% and 16.5%) [16], Nigeria (31.1%) [17], South Africa (27%–57.9% and 3%–57.8%) [5, 18, 19], Thailand (32%–33% and 7%–14%) [5, 20], Uganda (34.1% and 23.4%) [21] and Venezuela (32% and 15%) [5]. A 2010 study on Vietnamese medical students showed a rate of problem drinking at 12.3% [22].

Various factors have been identified to be associated with heavy drinking among university students, including (1) socio-demographic factors such as male gender [5, 11, 15], age, religious affiliation, national per capita alcohol consumption, income, living away from home [5, 7, 11, 14, 15, 23, 24]; (2) knowledge about health-related consequences [5] and attitudes towards alcohol use [5, 24] and (3) health-related variables such as tobacco use [11, 15, 25], illicit drug use [11, 26], gambling [27], high physical activity [11] and low life satisfaction [28, 29]. Many of these factors were evident in Asian countries [22, 30].

Heavy drinking among university students has many negative ramifications. For instance, a study on Australian students unveils a high level of exposure to alcohol-related harms, such as drink-driving, interpersonal aggression, social nuisance, inadequate security, sexually risky behavior and physical malaise [31]. A study in Vietnam depicts that university students who drank heavily suffered ‘loss of control, acute consequences and withdrawal’ and ‘negative influence on daily activities’ [32]. A study on Asian undergraduates at American colleges reveals that heavy episodic drinking was correlated with poor academic performance [33]. A multi-country study, including some Asian nations, shows a high prevalence of alcohol use and its association with psychological distress (anxiety-induced sleeplessness and/or depression) among adolescent students [34]. Thus, comprehending social and health factors associated with heavy drinking among university students can improve anti-alcohol interventions that tackle alcohol-related harm and enhance scholastic achievement among this population.

In the Association of South-East Asian Nations (ASEAN) region, studies on binge drinking among university students have been scant, country-specific and in different time frames. These limitations bar direct comparisons on common social and health attributes of heavy drinking among the student population in these countries. Our study was the first attempt to examine this issue across the region covering nine countries over the same period, using a standard questionnaire that allowed for direct comparisons. The aim of this study was to explore the prevalence of binge drinking and identify social and health factors associated with it among university students in low, middle-income and emerging economy countries in the ASEAN region.

## Methods

### Study design and participants

This multi-country, cross-sectional study was conducted in 2014 and 2015 among 8995 undergraduate students from nine ASEAN countries, of whom 62.3% were female, with a mean age of 20.5 years (SD = 2.0). This study was conducted as part of a larger investigation on health behaviors among university students by a network of researchers in Cambodia, Indonesia, Laos, Malaysia, Myanmar, the Philippines, Singapore, Thailand and Vietnam. Convenient sampling method was used to select universities for the survey, considering the complexity of the multi-site nature of the study as well as resource and time constrains. In Cambodia, Indonesia and Thailand, the survey was conducted at two universities in each country. In the rest, the survey was done at one university in each country.

### Data collection and sampling procedures

A structured questionnaire was developed in English, and then translated and back-translated into the national languages (Bahasa, Khmer, Burmese, Laotian, Thai and Vietnamese) of the participating countries. Research assistants, who were trained for 2 days specifically for this survey, administered the questionnaire in classrooms selected through a stratified random sampling procedure. One department was randomly selected from each faculty as a primary sampling unit, and from each selected department, students were randomly selected from all the courses. The selected students self-administered the questionnaire in the classrooms. Participation rates in most of the participating countries were more than 90%, except for Indonesia (86%) and Myanmar (73%).

### Variables and measurement

This study employed different measures adapted from previous studies to assess alcohol use and related health and social variables. Binge alcohol use was assessed using a question, “How often do you have (for men) five or more and (for women) four or more drinks on one occasion?” Response options were 0 = never, 1 = less than monthly, 2 = monthly, 3 = weekly and 4 = daily or almost daily [35]. According to the WHO, binge drinking (or heavy episodic drinking) “is defined as drinking at least 60 grams or more of pure alcohol on at least one occasion in the past 30 days” [36]. In our study countries, a standard unit of alcoholic beverage contains 12 g of pure alcohol, which means 5 units make 60 g in men. For women, the threshold is lower, here 4 standard drinks, in total at least 48 g.

Knowledge of alcohol effects was measured using two yes/no questions, “Do you believe that heart disease is influenced by alcohol use?” and “Do you believe that high blood pressure is influenced by alcohol use?” [37]. Health belief in alcohol drinking was assessed by asking the students to rate their perception regarding the importance of not drinking too much alcohol. The response options ranged from (1) of very low importance to (10) of very high importance [37].

Tobacco use was assessed using a yes/no question, “Do you currently use one or more of the following tobacco products (cigarettes, snuff, chewing tobacco, cigars, etc.)?” [38]. Illicit drug use was assessed using a question, “How often have you taken drugs, other than those prescribed by a health care provider, in the past 12 months?” with response options of: (1) 0 time, (2) 1–2 times, (3) 3–9 times and (4) 10 or more times.

Subjective general health status was assessed using a question, “In general, would you say your health is…?” with response options ranging from (1) excellent to (5) poor [37]. Life satisfaction was assessed using a question, “All things considered, how satisfied are you with your life as a whole?” with response options ranging from (1) very satisfied to (5) very dissatisfied [37].

Ten items of the Centre for Epidemiologic Studies Depression Scale (CES-D) were used to assess depressive symptoms; and an individual with a score of ≥15 would be classified as having severe depressive symptoms [39]. Cronbach’s alpha in this study was 0.73. Physical activity was assessed using the self-administered International Physical Activity Questionnaire (IPAQ) short version that collected physical activity data in the past 7 days (IPAQ-S7S). We used the instructions given in the IPAQ’s manual for reliability and validity, which is detailed elsewhere [40]. We categorized physical activity according to the official IPAQ’s scoring protocol (IPAQ 2006) as low, moderate and high.

Religiousness was assessed using two items of the five-item Duke University Religion Index (DUREL) [41] focusing on organized and non-organized religious activities [42]. We asked, (1) “How often do you attend church or other religious meetings?” with response options ranging from (1) Never to (6) more than once per week, and (2) “How often do you spend time in private religious activities such as prayer, meditation or Bible study?” with response option ranging from (1) rarely or never to (6) more than once per day. Socio-demographic questions included age, gender, residential status and subjective socioeconomic background.

### Data analyses

To address gender differences, data were analyzed separately for male and female students. Descriptive statistics were used for reporting the proportion of binge drinking. Chi-square test was used for categorical variables and Student’s *t*-test was used for continuous variables to compare the prevalence of binge drinking in socio-demographic characteristics as well as social and health behaviors (health beliefs in alcohol drinking general health status, mental health, other substance use, physical activity, religiousness).

Two multivariable logistic regression models (one for male and another one for female students) were constructed to explore social and behavioral factors associated with any binge drinking. All variables significantly associated with binge drinking in bivariate analyses at a level of *p-*value < 0.05 were simultaneously included in the models. Crude and adjusted odds ratio (AOR) were obtained and reported with confidence intervals (CI) and *p*-values. STATA 11.00 (StatCorp LP, College Station, TX) was used for all statistical analyses.

### Ethics statement

The study was approved by an ethical committee in each country. A written or verbal consent was obtained from each participant after they were made clear that participation in the study was voluntary, and they had an opportunity to refuse or discontinue their participation at any time and for any reason. Confidentiality of the data and privacy of the participants were protected by administering the questionnaires in a private premise and excluding personal identifiers in the survey.

## Results

Table 1 shows prevalence of binge drinking among male and female students. Of the total sample across the nine countries, 80.8% were non-binge drinkers, while 12.8% were infrequent (less than once per month) and 6.4% were frequent (at least once per month) binge drinkers. The overall prevalence of any (infrequent or frequent) binge drinking differed by country, ranging from below 2.5% in Indonesia and Malaysia to 39.1% in Thailand and 55.0% in Laos. Male students were significantly more likely to be frequent binge drinkers than females (10.1% vs. 4.2%, *p* < 0.001). Although the preponderance of binge drinking among male students was true for students from eight countries, there was no significant gender difference in binge drinking rate in Malaysia.

**Table 1:** Prevalence of binge drinking among male and female university students from nine countries in ASEAN

Country		Total sample			Male			Female				
		Binge drinking			Binge drinking			Binge drinking				
	Total	Never	Infrequent	Frequent	Never	Infrequent	Frequent	Never	Infrequent	Frequent	χ^2^ (df)	*P*-value
Cambodia^a^	1357	91.7	4.3	4.1	85.1	7.4	7.5	99.5	1.0	0.4	80.6 (2)	<0.001
Indonesia^a^	981	98.5	0.8	0.7	96.9	1.7	1.4	99.1	0.4	0.4	70.1 (2)	0.023
Laos^a^	806	44.9	30.6	24.4	22.7	31.1	46.2	56.3	30.4	13.3	125.0 (2)	<0.001
Malaysia^b^	1023	97.7	2.1	0.3	96.8	3.0	0.2	98.5	1.2	0.4	4.5 (2)	0.105
Myanmar^a^	491	90.0	8.6	1.4	80.9	5.8	3.3	96.8	3.2	0.0	35.1 (2)	<0.001
Philippines^a^	782	88.7	54.3	6.6	78.6	8.0	13.4	92.3	4.0	3.8	30.1 (2)	<0.001
Singapore^c^	894	71.4	22.3	6.4	62.8	28.7	8.5	79.9	15.8	4.3	37.7 (2)	<0.001
Thailand^b^	1658	60.9	28.3	10.8	43.2	34.4	22.4	65.0	26.9	8.1	71.8 (2)	<0.001
Vietnam^a^	817	93.3	5.3	1.5	88.4	8.9	2.7	98.1	1.7	0.2	30.8 (2)	<0.001
All	8809	80.8	12.8	6.4	75.6	14.3	10.1	83.9	11.9	4.2	138.3 (2)	<0.001

*Notes: Infrequent ≤ monthly; Frequent = Monthly/ Weekly/daily or almost daily*

*Abbreviation: ASEAN association of Southeast Asian Nations; df degree of freedom*

^a^Lower-middle income country; ^b^Upper-middle income country; ^c^High-income country

As shown in Table 2, prevalence of binge drinking was significantly higher among male students in the age group of 20 to 21 (22.5%) and 22 and older (34.3%) compared to 12.9% among students in the age group of 18–19. In female students, prevalence was 11.6%, 19.4% and 17.8% in students in the age group of 18 to 19, 20 to 21 and 22 and older, respectively. Compared to those living with parents or guardians, prevalence of binge drinking was significantly lower among male students who were not living with parents or guardians (24.5% vs. 21.3%). In contrast, prevalence of binge drinking was significantly higher among female students who were not living with parents or relatives (19.7% vs. 14.8%). Prevalence of binge drinking was significantly lower among female students from a wealthy or quite well-off family compared to those from a poor or not well-off family (18.3% vs. 12.2%). In both males and females, prevalence of binge drinking was significantly higher among students living in an upper-middle- or high-income country compared to those living in a lower-income country (28.4% vs. 22.0% among males, 24.7% vs. 9.8% among females). Regarding religiosity, prevalence of binge drinking was significantly lower among male and female students with high level of involvement compared to those with low level of involvement in both organized and non-organized religious activity (Table 2).

**Table 2:** Comparison of prevalence of binge drinking in different characteristics among male and female university students in nine ASEAN countries

Variables	Male (*n* = 3324)		Female (*n* = 5482)	
	Number (%)	OR (95% CI)	Number (%)	OR (95% CI)
*Socio-demographic characteristics*				
Age groups				
18–19	107 (12.9)	Reference	242 (11.6)	Reference
20–21	291 (22.5)	1.95 (1.53–2.49)***	409 (19.4)	1.83 (1.54–2.17)***
22 or older	413 (34.3)	3.52 (2.78–4.45)***	230 (17.8)	1.64 (1.35–2.00)***
Living situation				
Not with parents/guardians	412 (24.5)	Reference	642 (19.7)	Reference
With parents/guardians	296 (31.3)	0.71 (0.60–0.85)***	229 (14.8)	1.41 (1.20–1.67)***
Family economic status				
Not well off/poor	502 (25.4)	Reference	639 (18.3)	Reference
Wealthy/quite well off	309 (23.0)	0.88 (0.75–1.04)	242 (12.2)	0.62 (0.53–0.73)***
Country category				
Upper-middle/high income	358 (28.4)	Reference	570 (24.7)	Reference
Lower-middle income	453 (22.0)	0.71 (0.60–0.83)***	311 (9.8)	0.33 (0.29–0.39)***
Level of involvement in organized religious activity				
Low	258 (30.9)	Reference	146 (16.7)	Reference
Medium	317 (34.2)	1.16 (0.95–1.42)	600 (24.1)	1.58 (1.30–1.93)***
High	130 (15.2)	0.40 (0.32–0.51)***	122 (8.5)	0.47 (0.36–0.60)***
Level of involvement in non-organized religious activity				
Low	489 (33.3)	Reference	465 (22.9)	Reference
Medium	127 (29.1)	0.82 (0.65–1.04)	285 (24.7)	1.11 (0.93–1.31)
High	89 (12.4)	0.28 (0.22–0.36)***	116 (7.2)	0.26 (0.21–0.32)***
*Health knowledge and beliefs*				
Knowledge of alcohol-heart disease relationship	327 (22.8)	0.63 (0.53–0.74)***	375 (14.9)	0.62 (0.54–0.72)***
Knowledge of alcohol-high blood pressure relationship	407 (26.1)	0.88 (0.73–1.04)	555 (19.5)	1.24 (1.06–1.44)**
Weak beliefs in importance of limiting alcohol use	7.4 (2.7)	0.85 (0.82–0.87)***	7.8 (2.5)	0.82 (0.80–0.85)***
Subjective health status (range 1–5)	2.9 (1.0)	0.88 (0.82–0.95)**	3.1 (0.9)	1.05 (0.96–1.14)
Level of life satisfaction				
Low	421 (37.3)	Reference	526 (24.1)	Reference
Medium	236 (20.2)	0.43 (0.35–0.51)***	238 (12.1)	0.44 (0.37–0.52)***
High	50 (14.9)	0.30 (0.21–0.41)***	105 (16.1)	0.60 (0.48–0.76)***
Current tobacco use	118 (51.1)	3.65 (2.78–4.79)***	62 (47.7)	5.21 (3.67–7.42)***
Past year illicit drug use	189 (42.2)	3.19 (2.58–3.95)***	206 (23.1)	2.37 (1.97–2.86)***
Depressive symptoms	91 (28.5)	1.14 (1.08–1.68)*	105 (19.8)	1.76 (1.34–2.33)***
Physical activity				
Low	313 (22.6)	Reference	461 (15.6)	Reference
Medium	241 (20.9)	0.91 (0.75–1.10)	254 (14.4)	0.91 (0.77–1.07)
High	247 (32.5)	1.65 (1.35–2.01)***	147 (21.5)	1.49 (1.21–1.83)***

*Abbreviation: ASEAN association of Southeast Asian Nations, CI confidence interval, OR odds ratio*

*Note: Values are number (%) for categorical variables and mean (standard deviation) for continuous variables*

*P < 0.05, **P < 0.01, ***P < 0.001

Table 2 also shows that prevalence of binge drinking was significantly lower among male and female students with better knowledge on relationship between alcohol and heart disease and among female students with knowledge on relationship between alcohol and high blood pressure. The prevalence was also significantly higher among students with weak beliefs in the importance of limiting alcohol use in both gender and lower score of subjective health status in male students. In both males and females, prevalence of binge drinking was significantly lower among students with higher level of life satisfaction, higher among those who reported using other substances such as tobacco and illicit drugs and those who reported depressive symptoms. Interestingly, prevalence of binge drinking was significantly higher among both male and female students who reported high level of physical activity compared to those with low level of physical activity.

Table 3 shows results from multivariate logistic regression analyses stratified by gender. After adjustment, among males, higher prevalence of binge drinking remained significantly associated with being in older age group of 20–21 (AOR = 1.83, 95% CI = 1.54–2.17) and 22 or older (AOR = 4.67, 95% CI = 3.30–2.00), living with parents or guardians (AOR = 0.69, 95% CI = 0.55–0.86), lower level of non-organized religious activity (AOR = 0.29, 95% CI = 0.19–0.44), lack of knowledge on alcohol-heart disease relationship (AOR = 0.79, 0.64–0.98), weak beliefs in the importance of limiting alcohol use (AOR = 0.89, 95% CI = 0.85–0.93), poor subjective health status (AOR = 0.92, 95% CI = 0.72–0.99), lower level of life satisfaction (AOR = 0.46, 95% CI = 0.30–0.72), use of other substances such as tobacco (AOR = 3.50, 95% CI = 2.50–4.91) and illicit drug (AOR = 4.91, 95% CI = 2.24–3.87), depressive symptoms (AOR = 1.58, 95% CI = 1.10–2.27) and high level physical activity (AOR = 1.50, 95% CI = 1.15–1.97).

**Table 3:** Factors associated with any binge drinking in multivariable analyses among male and female university students in nine ASEAN countries

Variables in the model	Male	Female
	AOR (95% CI)^†﻿^	AOR (95% CI)^‡﻿^
Age groups		
18–19	Reference	Reference
20–21	2.30 (1.61–3.28)***	1.46 (1.15–1.85)**
22 or older	4.67 (3.30–6.59)***	1.86 (1.44–2.41)***
Living situation		
Not with parents/guardians	Reference	Reference
With parents/guardians	0.69 (0.55–0.86)***	1.12 (0.87–1.45)
Family economic status		
Not well off/poor		Reference
Wealthy/quite well off		0.61 (0.49–0.77)***
Country category		
Upper-middle/high income	Reference	Reference
Lower-middle income	0.88 (0.69–1.11)	0.37 (0.29–0.47)***
Level of involvement in organized religious activity		
Low	Reference	Reference
Medium	1.30 (0.96–1.68)	1.42 (1.10–1.83)**
High	0.72 (0.48–1.07)	0.85 (0.60–1.21)
Level of involvement in non-organized religious activity		
Low	Reference	Reference
Medium	0.95 (0.67–1.33)	1.19 (0.92–1.54)
High	0.29 (0.19–0.44)***	0.35 (0.25–0.48)***
Knowledge of alcohol-heart disease link	0.79 (0.64–0.98)*	0.67 (0.56–0.82)***
Knowledge of alcohol-high blood pressure link		1.22 (1.01–1.49)*
Weak beliefs in importance of limiting alcohol	0.89 (0.85–0.93)***	0.84 (0.81–0.88)***
Subjective health status	0.92 (0.72–0.99)*	
Level of life satisfaction		
Low	Reference	Reference
Medium	0.54 (0.42–0.69)***	0.53 (0.42–0.67)***
High	0.46 (0.30–0.72)***	0.71 (0.51–0.99)*
Current tobacco use	3.50 (2.50–4.91)***	6.01 (3.89–9.29)***
Past year illicit drug use	2.94 (2.24–3.87)***	2.58 (2.05–3.25)***
Depressive symptoms	1.58 (1.10–2.27)*	1.38 (1.02–1.87)*
Physical activity		
Low	Reference	Reference
Medium	0.99 (0.76–1.29)	1.06 (0.85–1.32)
High	1.50 (1.15–1.97)**	1.38 (1.06–1.79)*

*Abbreviations: AOR adjusted odds ratio, CI confidence interval; COR crude odds ratio*

*P < 0.05; **P < 0.01; ***P < 0.001

^†^ *Hosmer & Lemeshow Chi-square = 13.57, P = 0.09; Nagelkerke R* ^*2*^ *= 0.31*

^‡^ *Hosmer & Lemeshow Chi-square = 26.64, P < 0.001; Nagelkerke R* ^*2*^ *= 0.25*

Among females, higher prevalence of binge drinking remained significantly associated with being in the older age group of 20–21 (AOR = 1.46, 95% CI = 1.15–1.85) and 22 or older (AOR = 1.86, 95% CI = 1.44–2.41), poorer family background (AOR = 0.61, 95% CI = 0.49–0.77), living in an upper-middle- or high-income country (AOR = 0.37, 95% CI = 0.29–0.47), lower level of non-organized religious activity (AOR = 0.35, 95% CI = 0.25–0.48), lack of knowledge on alcohol-heart disease relationship (AOR = 0.67, 95% CI = 0.56–0.82), lack of knowledge on alcohol-high blood pressure relationship (AOR = 1.22, 95% CI = 1.01–1.49), weak beliefs in the importance of limiting alcohol use (AOR = 0.84, 95% CI = 0.81–0.88), lower level of life satisfaction (AOR = 0.53, 95% CI = 0.42–0.67), use of other substance use such as tobacco (AOR = 6.01 (3.89–9.29) and illicit drug (AOR = 2.58, 95% CI = 2.05–3.25), depressive symptoms (AOR = 1.38, 95% CI = 1.02–1.87) and high level of physical activity (AOR = 1.38, 95% CI = 1.06–1.79).

## Discussion

This study identified social and health determinants of binge drinking among university students in nine ASEAN countries. We found that 12.8% and 6.4% of the participants were respectively infrequent and frequent binge drinkers. These figures are lower than those in a study in 24 countries across Asia, Africa and the Americas, which found 17.1% and 11.3% of university students were respectively moderate and heavy alcohol drinkers [43].

The overall prevalence of binge drinking among university students ranged from below 2.5% in Indonesia and Malaysia to 39.1% in Thailand and 55.0% in Laos. These differences may be explained by students’ religious affiliations. Muslim countries (such as Indonesia and Malaysia) restrict alcohol consumption more strictly than Buddhist countries (such as Thailand and Laos) whose general population consumes more alcohol. Thailand has implemented laws that prohibit the sale of alcohol to children under the age of 18 years, and the sale of alcohol around schools and universities. This finding confirms the global trend, which indicates that Muslim countries consume less alcohol than non-Muslim countries [1]. For instance, the Eastern Mediterranean region, which consists mostly of Islamic states, has quite higher rates of abstinence and extremely lower rates of alcohol use disorders and alcohol dependence than other regions. The region had the abstinence rate of 94.6% in 2010, the average alcohol per capita consumption of 0.7 l during 2008–2010 and the prevalence rates of alcohol use disorders and alcohol dependence of 0.3% and 0.2%, respectively in 2010. In contrast, the European region had the abstinence rate of 33.6% in 2010, the average alcohol per capita consumption of 10.9 l during 2008–2010 and the prevalence rates of alcohol use disorders and alcohol dependence of 7.5% and 4.0%, respectively in 2010. The minimal alcohol drinking in Muslim countries is attributable to the religious restriction and subsequently strict alcohol control, especially the highest prevalence of regulations on days of sales off-premise.

Among the ASEAN countries, Thailand has the best regulatory framework to curb booze consumption [2]. Under its Alcohol Control Act, bars, clubs and retailers are prohibited from selling alcoholic beverages within a 300-m radius of higher-educational institutions. Also, retailers are banned from selling alcohol between midnight and 11 am and between 2 pm and 5 pm. This national alcohol policy persuaded more than two-thirds of Thais to stop drinking by 2015; and alcohol consumption in Thailand plateaued after the 2008 release of this Act. Vietnam, the Philippines and Indonesia have also introduced policies over the past few years to sap alcohol demand. Thailand and the Philippines have lofty taxes on alcohol that make it pricey to discourage youth from drinking. Myanmar has no national policy or action plan to tackle alcohol and there is no legal requirement that alcohol advertisements and containers carry health warnings [1]. A review on alcohol policies and alcohol consumption in low- and middle-income countries uncovered that alcohol policies regulating the physical availability of alcohol, particularly those concerning business hours or involving a licensing system for off-premises alcohol retail sales, as well as minimum legal drinking age, were associated with lower alcohol consumption [44].

In line with other studies [5, 11, 15, 24, 43], this study found that men and older age were associated with higher prevalence of binge drinking. The heavy drinking among male university students could be largely explained by a generic fact that compared to women, men are less often abstainers and drink more frequently and in larger quantities [1]. Concerning older age, early initiation of alcohol use could be a predictor of binge drinking for older adults. Research in other regions shows that people who start drinking at a young age tend to have an increased risk for alcohol dependence and abuse at later ages [45–47].

The association between male students’ binge drinking and living with parents or guardians signifies the role of parents or guardians in influencing children’s drinking behaviors. Parents’ alcohol use and attitudes toward use determine children’s drinking patterns. A study in the United States indicated that about two-thirds of college students who binge drank reported that their parents drank while they were growing up, and only about one-third of them reported that their parents disapproved of drinking while they were growing up [48]. Moreover, a family history of alcohol use disorders is a major environmental factor for vulnerability to alcohol abuse by children [49, 50]. Parents with alcohol use disorders increase the likelihood of their children developing risky drinking patterns when they grow up.

Moreover, female students with poorer family background and living in an upper-middle income or high-income country determined heavy drinking. These findings are similar to those in research elsewhere that pointed out high income [23] as a predictor of problem drinking. This is consistent with the global pattern, which depicts that greater economic wealth is associated with higher levels of alcohol consumption and lower abstention rates [1]. Specifically, among higher socioeconomic groups there are more drinkers and drinking occasions that induce problem drinking, while abstainers are more prevalent in the poorest groups. The link between binge drinking and poorer family background could be explained that rising wealth has increased the availability and affordability of alcohol and thus at-risk drinking, particularly to lower-income groups in growing economies [51, 52].

The relationship between binge drinking and the lack of non-organized religious activity or adherence to religious affiliation reflects the role of culture in relation to alcohol use [15]. Cultural and religious beliefs and norms can influence drinkers’ attitude to and perception about alcohol consumption, and thus can shape their drinking pattern and vulnerability to alcohol-related harm [1]. A previous study found that students who up took binge drinking in college were less likely than their non-uptake peers to report that religion was important to them [48]. Studies show that alcohol use and alcohol-related harm vary among drinkers with different ethnicities or religious affiliations [53–56]. To exemplify, as afore-mentioned, Muslim countries have the lowest rates of alcohol consumption, alcohol use disorders and alcohol dependence [1].

Lack of risk awareness of alcohol-heart disease link and weak beliefs in the importance of limiting alcohol use were also related with binge drinking. These confirm that lack of knowledge about health-related consequences [5, 43] and negative attitudes towards alcohol use [5, 24, 33, 43] were associated with heavy drinking. College students who heavily drank were more likely than their non-uptake peers to report inflated definitions of binge drinking. Further, these binge drinkers were more likely to report a normative perception that they drank because other people drank.

Finally, poor life satisfaction, other substance use (tobacco and illicit drug), depressive symptoms and high physical activity were associated with binge drinking. Again, these results iterate previous findings in other studies [11, 15, 25, 26, 28, 29, 43]. These findings could be explained that in general relationships between problem drinking, other substance use and quality of life are intertwined and often mutually compounding.

### Study strengths and limitations

The strengths of this multi-country study include the large sample size recruited from 13 universities in nine countries in the region with different cultures and socio-demographic characteristics and the use of standardized methods and tools across the study sites.

This study also had several limitations. First, the main variables, such as alcohol and other substance use, were self-reported. Given the persistent stigma related to substance use in Asian cultures, these behaviors could be underreported. However, self-reported measures are an important source of information, considering the scarcity of data on these behaviors, especially in Asian youth. Second, the generalizability of the study findings is questionable given that the data were collected from university students in one or two cities in each participating country. Therefore, the extent to which these results may be generalized to students in other areas of those countries or other parts of ASEAN remains unknown. The final limitation concerns the cross-sectional nature of the study, which limited our ability to establish causality. Longitudinal studies concerning substance use beginning in early adolescence are necessary for future research in the region.

## Conclusions

This study uncovered that 18.8% of university students in the nine ASEAN countries were binge drinkers. Age, gender, living arrangement, economic status, religious activity, related health knowledge, beliefs in and attitudes toward alcohol use, life satisfaction, use of other substances, depressive symptoms and physical activity were the chief health and social correlates of binge drinking among the students. These findings suggest the importance of a comprehensive intervention approach to prevent the acquisition of binge drinking in this population. University health promotion programs that integrate binge drinking, other substance use, co-occurring addictive behaviors and health beliefs should be implemented in the respective countries.

## Abbreviations

AOR: Adjusted odds ratio
ASEAN: Association of Southeast Asian Nations
CES-D: Centers for epidemiologic studies depression scale
CI: Confidence interval
DUREL: Duke University Religion index
IPAQ: International physical activity questionnaire
SD: Standard deviation
